# Hydroxysafflower Yellow A Inhibits Vascular Adventitial Fibroblast Migration via NLRP3 Inflammasome Inhibition through Autophagy Activation

**DOI:** 10.3390/ijms24010172

**Published:** 2022-12-22

**Authors:** Lin Liu, Qingzhuo Cui, Junna Song, Yang Yang, Yixin Zhang, Jiapeng Qi, Jingshan Zhao

**Affiliations:** 1 College of Pharmacy, Hebei University of Chinese Medicine, Shijiazhuang 050200, China; 2 Department of Biochemistry and Molecular Biology, College of Basic Medicine, Hebei University of Chinese Medicine, Shijiazhuang 050200, China; 3 Traditional Chinese Medicine Processing Technology Innovation Center of Hebei Province, College of Pharmacy, Hebei University of Chinese Medicine, Shijiazhuang 050200, China

**Keywords:** hydroxysafflower yellow A, vascular adventitial fibroblasts, NLRP3 inflammasome, autophagy

## Abstract

Inflammation is closely associated with progression of vascular remodeling. The NLRP3 inflammasome is the key molecule that promotes vascular remodeling via activation of vascular adventitia fibroblast (VAF) proliferation and differentiation. VAFs have a vital effect on vascular remodeling that could be improved using hydroxysafflower yellow A (HSYA). However, whether HSYA ameliorates vascular remodeling through inhibition of NLRP3 inflammasome activation has not been explored in detail. Here, we cultured primary VAFs and analyzed the migration of VAFs induced by angiotensin II (ANG II) to determine the potential effects and mechanism of HSYA on VAF migration. The results thereof showed that HSYA remarkably inhibited ANG II-induced VAF migration, NLRP3 inflammasome activation, and the TLR4/NF-κB signaling pathway in a dose-dependent manner. In addition, it is worth noting that LPS promoted ANG II-induced VAF migration and NLRP3 inflammasome assembly, which could be significantly reversed using HSYA. Moreover, HSYA could be used to inhibit NLRP3 inflammasome activation by promoting autophagy. In conclusion, HSYA could inhibit ANG II-induced VAF migration through autophagy activation and inhibition of NLRP3 inflammasome activation through the TLR4/NF-κB signaling pathway.

## 1. Introduction

Cardiovascular disease (CVD) is the leading death-causing disease in human beings due to an increasingly aging population, with more than 60 million lives lost to CVD in Europe annually, especially in the people who were 65 years of age or older [1]. In line with diet changes and sedentary lifestyles, CVD, which was once thought to only affect the elderly, is occurring in an increasing number of young people [2]. Vascular remodeling, which is the pathological basis of CVD, involves abnormal activation, proliferation, and migration of vascular wall cells [3]. A vascular wall can be divided into three layers: the adventitia, the media, and the tunica intima. Previously, numerous researchers have focused on the tunica intima and the media [4,5]. However, in contrast to the tunica intima and the media, the vascular adventitia, which is situated away from the lumen, has not been researched thoroughly enough. Recently, some studies have shown that the adventitia has a crucial effect on vascular remodeling [6,7]. Vascular wall inflammation is closely related to numerous CVDs and involves inflammatory cell infiltration into the vascular adventitia [8,9]. Vascular adventitia fibroblasts (VAFs), the uppermost cell type in the adventitia, participate in angiotensin-induced vascular remodeling and trigger interleukin-6 (IL-6) expression and inflammatory responses [7,10]. Fibroblasts activated by stimuli could recruit inflammatory cells, which would further activate fibroblasts to promote fibroblast proliferation, adventitial thickening, and additional cytokine secretion [7]. Thus, inhibition of inflammation responses and fibroblast proliferation may be a new strategy for CVD treatment.

The NLRP3 (nucleotide-binding domain, leucine-rich–containing family, pyrin domain–containing-3) inflammasome is a cytoplasmic multiprotein complex that consists of the sensor NLRP3, an apoptotic-associated speck-like adaptor protein with a caspase recruitment domain (ASC), and effector Caspase1. The NLRP3 inflammasome is capable of sensing tissue damage and promotes maturation and secretion of pro-inflammatory cytokines such as interleukins (IL-1β, IL-18, etc.). In addition, the NLRP3 inflammasome exerts essential effects on the migration and phenotypic transformation of VAF, which result in adventitial remodeling [11,12]. Reduction of NLRP3 inflammasome activation can inhibit transformation of VAF phenotypes and vascular remodeling and improve the symptoms of cardiac fibrosis and hypertrophy [13]. Autophagy, an evolutionarily conserved lysosomal degradation system, plays a vital role in preventing CVD [14] and is closely connected to NLRP3 inflammasome activation [15,16]. Through construction of an Atg7 knockout mouse model, Kim et al. found that autophagy activation can inhibit NLRP3 inflammasome activation and ameliorate steatohepatitis [17]. Additionally, autophagy can regulate the inflammatory responses of fibroblasts [18]. However, the relationship between autophagy and the NLRP3 inflammasome in fibroblasts has not been reported.

Hydroxysafflower yellow A (HSYA) is the main active compound of safflower, a traditional Chinese medicine. Li et al. showed that HSYA protected against hypoxia-induced pulmonary hypertension via reversal of remodeling of the pulmonary artery through inhibition of proliferation and hypertrophy of rat pulmonary-artery smooth-muscle cells [19]. Our previous study indicated that HSYA could be used to inhibit VAF migration by regulating autophagy [20]. However, whether HSYA inhibits NLRP3 inflammasome activation via autophagy activation to exert potent protective effects against VAF migration has not yet been determined.

In the present study, we investigated this issue using VAFs treated with ANG II, LPS, and rapamycin. We explored the effects of HSYA on cell migration in VAFs induced by ANG II or promoted with LPS and rapamycin to evaluate whether, and through which mechanism, use of HSYA could prevent vascular remodeling associated with ANG II-induced VAF migration.

## 2. Results

### 2.1. HSYA Inhibits ANG II-Induced VAF Migration

**Figure 1:**
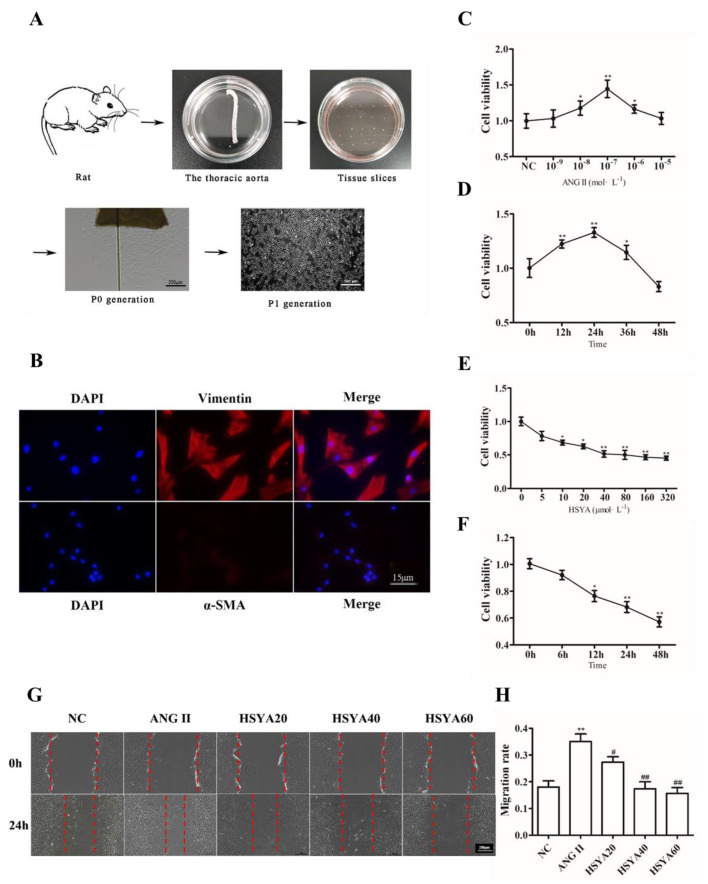
HSYA inhibits angiotensin II-induced VAF migration. (**A**) The primary culture protocol of VAFs. Scale bar, 500 µm. (**B**) Expressions of α-SMA and vimentin on VAFs separated from the adventitia of blood vessels (*n* = 3). Scale bar, 15 µm. Red represents vimentin or α-SMA; blue represents DAPI. (**C**) Effects of different concentrations of ANG II on the viability of VAF cells (*n* = 6). * Compared with the NC group, * *p* < 0.05, ** *p* < 0.01. (**D**) Effects of ANG II on VAF-cell viability at different times (*n* = 6). * Compared with the 0 h group, * *p* < 0.05, ** *p* < 0.01. (**E**) Effects of different concentrations of HSYA on the viability of VAF cells (*n* = 6). * Compared with the NC group, * *p* < 0.05, ** *p* < 0.01. (**F**) Effects of HSYA on VAF-cell viability of at different times (*n* = 6). * Compared with the 0 h group, * *p* < 0.05, ** *p* < 0.01. Data were obtained from three independent experiments. (**G**) Wound-healing assay of VAFs treated or untreated with ANG II and HSYA (*n* = 3). Scale bar, 200 µm. (**H**) The quantification of mobility of the VAFs in the five groups of (**G**). Data and images were obtained from three independent experiments. Data in (**G**) were analyzed via one-way ANOVA. * Compared with the NC group, # compared with the ANG II group, **^/##^ *p* < 0.05.

VAFs were successfully obtained from the adventitia of blood vessels. The cells were fibroblastoid and aggregated, consistent with the characteristics of fibroblasts (Figure 1A). To further verify the cell type, immunofluorescence staining was used to detect the expressions of vimentin and α-SMA, which are makers of fibroblasts and smooth muscle cells, respectively. As shown in Figure 1B, the expression of vimentin was positive with a negative expression of α-SMA, suggesting that the cells we obtained were fibroblasts (Figure 1B), which was further confirmed via Western blot (Supplementary Figure S1). The dose–response curve showed that the optimal concentrations of ANG II and HSYA were 100 nmol·L^−1^ and 40 µmol·L^−1^, respectively; the optimal treatment time for ANG II and HSYA was 24 h (Figure 1C–F). When the concentration and treatment time of HSYA were greater than or equal to 80 µmol·L^−1^ and 48 h, respectively, the VAFs showed morphological changes (Supplementary Figure S2). Thus, the optimal concentration and treatment times of HSYA were 40 µmol·L^−1^ and 24 h, respectively. Then, the VAFs were treated using ANG II and HSYA to determine whether HSYA could be used to inhibit ANG II-induced VAF migration. The VAFs were divided into an NC group, an ANG II group (10^−7^ mol·L^−1^), an HSYA20 group (10^−7^ mol·L^−1^ of ANG II + 20 µmol·L^−1^ of HSYA), an HSYA40 group (10^−7^ mol·L^−1^ of ANG II + 40 µmol·L^−1^ of HSYA), and an HSYA60 group (10^−7^ mol·L^−1^ of ANG II + 60 µmol·L^−1^ of HSYA). Compared with the NC group, the VAF migration rate was significantly higher in the ANG II group (Figure 1G,H). However, the VAF migration rate in the HSYA group was dose-dependently decreased compared to that in the ANG II group (Figure 1G,H). Thus, our data suggested that HSYA could be used to dose-dependently inhibit ANG II-induced VAF migration.

### 2.2. HSYA Inhibits ANG II-Induced NLRP3 Inflammasome Activation

**Figure 2:**
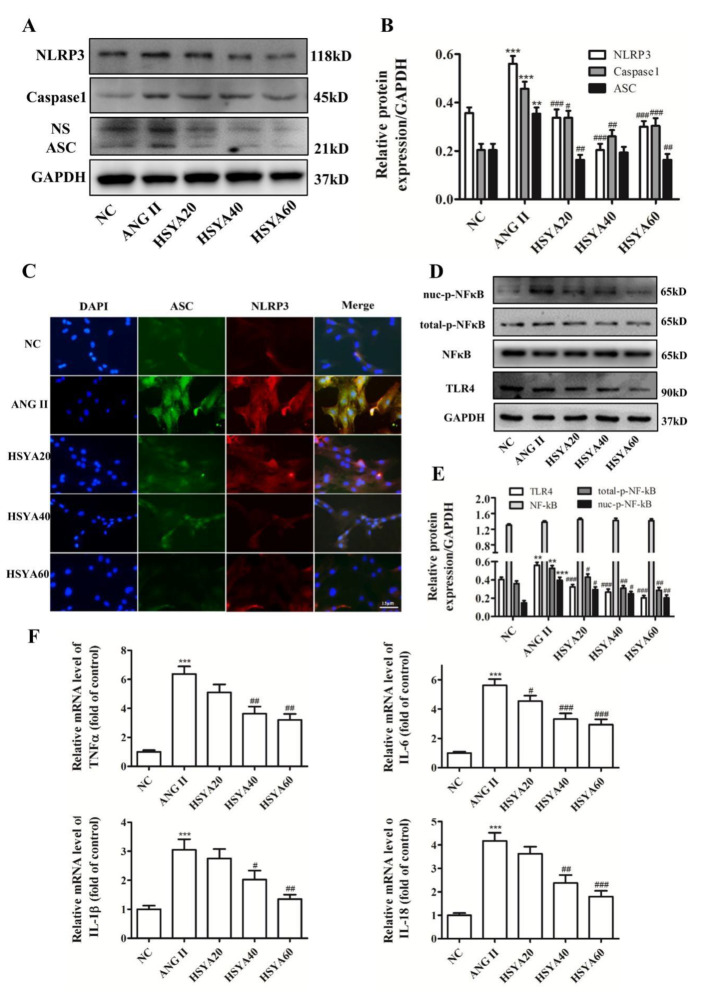
HSYA inhibits NLRP3 inflammasome activity through the TLR4/NF-κB signaling pathway. (**A**) The expressions of NLRP3 inflammasome-related proteins analyzed via Western blot. (**B**) The quantification of the protein blots in (**A**). (**C**) NLRP3 (red) and ASC (green) protein expression in VAFs (*n* = 3). Scale bar, 15 µm. (**D**) Western-blot analysis of the expression of TLR4/NF-κB-related proteins treated with ANG II and HSYA (*n* = 3). (**E**) The quantification of the protein blots in (**D**). (**F**) mRNA levels of IL-6, TNFα, IL-1β, and IL-18 in the five VAF groups were determined via quantitative real-time PCR. * Compared with the NC group, # compared with the ANG II group, ^#^ *p <* 0.05, *^##^ p* < 0.01, ***^/*###*^ *p <* 0.001.

Increasing experimental and clinical evidence indicates that inflammation exerts an important effect on cardiovascular disease occurrence, development, and complication. NLRP3 inflammasome overactivation leads to chronic vascular inflammation and is a common pathway for vascular remodeling, with various causes [21,22,23]. To explore the role of HSYA in NLRP3 inflammasome activation, we determined the expressions of NLRP3-inflammasome-associated proteins such as NLRP3, ASC, and Caspase1. Compared with the NC group, NLRP3-inflammasome-related protein expression was significantly higher in the ANG II group. However, compared with the ANG II group, the NLRP3, ASC, and Caspase1 protein expressions were significantly lower in the HSYA group (Figure 2A,B). Then, double immunofluorescence staining was applied to detect NLRP3 and ASC protein coexpression for NLRP3-inflammasome-assembly examination. As shown in Figure 2C, ANG II significantly increased the coexpression of NLRP3 and ASC; this was reversed with HSYA in a dose-dependent manner.

Next, we detected the level of protein expression in the TLR4/NF-κB signaling pathway, which is an upstream pathway of inflammasome activation [24]. Compared with the NC group, ANG II significantly increased the expressions of TLR4 and phosphorylated NF-κB (pNF-κB) protein (Figure 2D). Compared with the ANG II group, HSYA significantly decreased the protein expressions of TLR4 and total pNF-κB, as well as the expression of pNF-κB in the cell nucleus (Figure 2D,E). NLRP3 inflammasome activation promotes maturation and secretion of pro-inflammatory cytokines. Thus, the mRNA levels of pro-inflammatory cytokines IL-6, TNFα, IL-1β, and IL-18, which are activated by the NLRP3 inflammasome, were significantly higher in the ANG II group compared to those in the NC group (Figure 2F). However, HSYA remarkably decreased the levels of pro-inflammatory cytokines compared with those of the ANG II group (Figure 2F).

### 2.3. HSYA Inhibits ANG II-Induced VAF Migration through Inhibition of NLRP3 Inflammasome Activation

**Figure 3:**
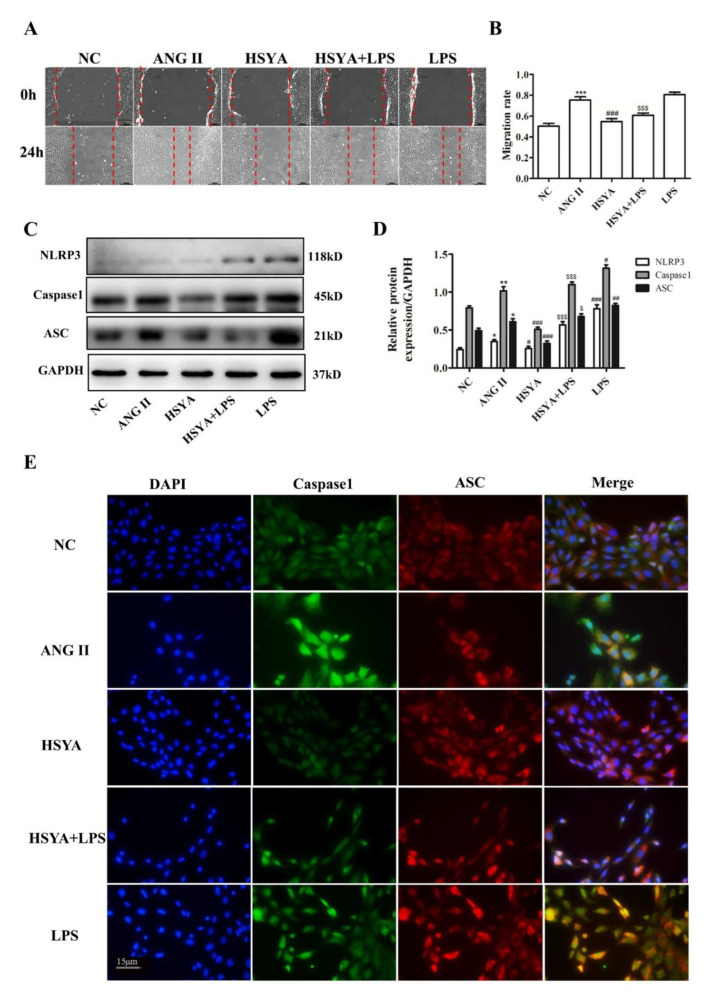
HSYA inhibited ANG II-induced VAF migration through inhibition of NLRP3 inflammasome activation. (**A**) Wound-healing assay of VAFs treated or untreated with ANG II, HSYA, and LPS (*n* = 3). Scale bar, 200 µm. (**B**) The quantification of the VAF mobility in the five groups of (**A**). (**C**) The expression of NLRP3-inflammasome-related proteins analyzed via Western blot. (**D**) The quantification of the protein blots in (**C**). (**E**) Effects of HSYA on LPS-induced coexpression of Caspase1 (green) and ASC (red) (*n* = 3). Scale bar, 15 µm. * Compared with the NC group, # compared with the ANG II group, $ compared with the LPS group, *^/#/$^ p < 0.05, **^/*##*^*p* < 0.01, ***^/*###/$$$*^ *p <* 0.001.

To further determine the HSYA mechanism that inhibits ANG II-induced VAF migration, the VAFs were treated with LPS, an NLRP3 inflammasome activator. The VAFs were divided into the NC group, the ANG II group (10^−7^ mol·L^−1^ of ANG II), the HSYA group (10^−7^ mol·L^−1^ of ANG II + 40 µmol·L^−1^ of HSYA), the HSYA + LPS group (10^−7^ mol·L^−1^ of ANG II + 40 µmol·L^−1^ of HSYA + 100 nmol·L^−1^ of LPS), and the LPS group (I 10^−7^ mol·L^−1^ of ANG I + 100 nmol·L^−1^ of LPS). Compared with the LPS group, the migration rate in the HSYA + LPS group decreased significantly, suggesting that HSYA reversed the promoting effect of LPS on VAF migration (Figure 3A,B). Next, we detected the expressions of NLRP3-inflammasome-related proteins such as NLRP3, Caspase1, and ASC. A Western blot assay showed that HSYA significantly decreased the expressions of NLRP3, Caspase1 and ASC compared with the LPS group (Figure 3C,D). We also detected the coexpression of ASC and Caspase1 to verify the effect of HSYA on NLRP3 inflammasome assembly. The results thereof indicated that compared to the ANG II group, the coexpression of ASC and Caspase1 significantly increased in the LPS group (Figure 3E). Amazingly, the coexpression of ASC and Caspase1 significantly decreased in the HSYA + LPS group compared to that in the LPS group (Figure 3E). Thus, our data suggested that HSYA could reverse NLRP3 inflammasome activation induced by ANG II and LPS.

### 2.4. HSYA Inhibits NLRP3 Inflammasome Activation through Promotion of Autophagy

**Figure 4:**
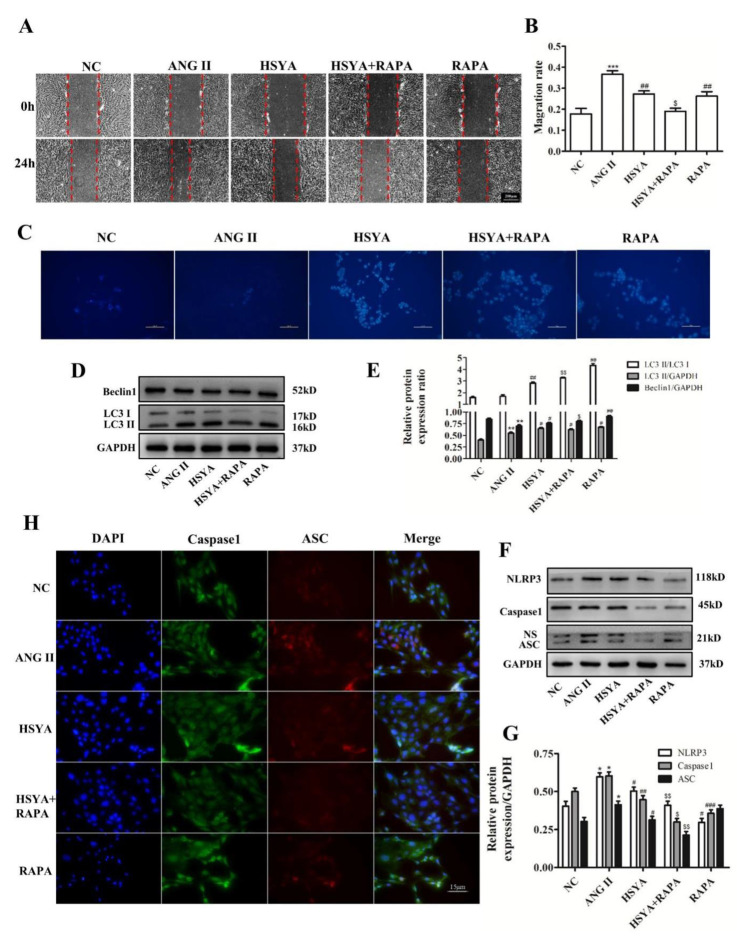
HSYA inhibits NLRP3 inflammasome activation via autophagy promotion. (**A**) Wound-healing assay of VAFs treated or untreated with ANG II, HSYA, and RAPA (*n* = 3). Scale bar, 200 µm. (**B**) The quantification of mobility of the VAFs in the five groups in (**A**). (**C**) The autophagosome (blue) was measured using MDC analysis (*n* = 3). Scale bar, 100µm. (**D**) Western-blot analysis of Beclin1 and LC3 (*n* = 4). (**E**) The quantification of the protein blots in (**D**). (**F**) Western-blot analysis of NLRP3-inflammasome-related proteins such as NLRP3, Caspase1, and ASC (*n* = 4). (**G**) The quantification of the protein blots in (**F**). (**H**) Double immunofluorescence staining analysis of the coexpressed Caspase1 (green) and ASC (red) proteins in VAFs (*n* = 4). Scale bar, 15 µm. * Compared with the NC group, # compared with the ANG II group, $ compared with the LPS group, *^/#/$^ p < 0.05, **^/*##/$$*^ *p* < 0.01, ***^/*###*^ *p <* 0.001.

Autophagy is a self-protective mechanism by which cells use lysosomes to degrade damaged proteins or organelles [25]. Our previous studies have shown that the effect of HSYA’s inhibition of VAF migration was related to autophagy [20]. Thus, we explored the relationship between NLRP3 inflammasome activation and autophagy. VAFs were divided into the NC group, the ANG II group (10^−7^ mol·L^−1^), the HSYA group (10^−7^ mol·L^−1^ of ANG II + 40 µmol·L^−1^ of HSYA), the HSYA + RAPA group (10^−7^ mol·L^−1^ of ANG II + 40 µmol·L^−1^ of HSYA + 100 nmol·L^−1^ of RAPA), and the RAPA group (10^−7^ mol·L^−1^ of ANG II + 100 nmol·L^−1^ of RAPA). A wound-healing assay indicated that both HSYA and rapamycin (RAPA) could more significantly inhibit VAF migration compared with that of the ANG II group (Figure 4A,B). A cotreatment of HSYA and RAPA further inhibited ANG II-induced VAF migration (Figure 4A,B). To further determine the autophagosome production, an MDC experiment was performed. The autophagy fluorescence spots in the HSYA group were significantly enhanced compared with those in the ANG II group. The spots in the HSYA + RAPA group were significantly enhanced compared with those in the RAPA group. The MDC experiment results indicated that either HSYA or RAPA could increase autophagy-fluorescence-spot intensity (Figure 4C). LC3 and Beclin1, downstream molecules of the autophagy signaling pathway, were detected via Western blot. We observed that the LC3 II and Beclin1 levels in the cells treated with HSYA and RAPA were significantly upregulated (Figure 4D,E). Next, expression levels of NLRP3-inflammasome-related proteins were determined to confirm the relationship between NLRP3 inflammasome assembly and autophagy activation. The results thereof indicated that when VAFs were treated with HSYA and RAPA, the expressions of the NLRP3, ASC, and Caspase1 proteins were significantly lower compared with those in the ANG II group (Figure 4F,G). Then, we detected a coexpression of Caspase1 and ASC using double immunofluorescence staining. We found that HSYA or RAPA could significantly decrease coexpression of Caspase1 and ASC compared with the ANG II group (Figure 4H). In addition, the autophagy inhibitor Bafilomycin A1 (Baf) significantly promoted VAF migration; this could be reversed with HSYA (Supplementary Figure S3). Thus, our data suggested that HSYA inhibited ANG II-induced VAF migration via autophagy promotion through inhibition of NLRP3 inflammasome activation and assembly.

## 3. Discussion

In the present study, we investigated the effects of HSYA regarding inhibition of ANG II-induced VAF migration and the underlying molecular mechanism thereof. We found that: (1) HSYA inhibited ANG II-induced VAF migration in a dose-dependent manner; (2) HSYA could downregulate NLRP3 inflammasome activity through the TLR4/NF-κB signaling pathway; (3) The role of HSYA in inhibition of ANG II-induced VAF migration was related to NLRP3 inflammasome assembly suppression; (4) HSYA inhibited NLRP3 inflammasome activation by promoting autophagy.

Previous studies have indicated that common pathological bases of cardiovascular diseases, such as atherosclerosis, aortic dissection, and hypertension, are closely related to vascular remodeling [26,27]. Vascular remodeling is a chronic inflammatory response [28] that involves vascular-wall cell activation, proliferation, and migration, resulting in vascular structural dysfunction, such as vascular wall thickening and luminal stenosis [29]. VAFs, the “sentinels” of vascular injury, are activated in the early stages of vascular injury. Activated fibroblasts, which recruit inflammatory cells and are further activated by inflammatory molecules, become dysfunctional, proliferate, and migrate to the intima of blood vessels, resulting in vascular wall thickening [30]. Thus, inhibition of VAF activation and migration might be a new strategy used to improve vascular remodeling and treat cardiovascular diseases. Li et al. [31] observed direct evidence of VAF migration to the intima of carotid-artery balloon injuries in rats through LacZ tracing and proved that LacZ-labeled VAFs had migrated to the intima before intima hyperplasia. Decreasing inflammation, proliferation, and migration of fibroblasts can inhibit vascular-adventitia thickening and improve hypertension pathogenesis [11]. In this study, VAFs, identified via positive vimentin and negative α-SMA, were successfully cultured from the thoracic aortae of Sprague–Dawley rats, and HSYA was confirmed to significantly inhibit ANG II-induced VAF migration (Figure 1).

Next, we explored the effect of HSYA on NLRP3 inflammasomes in ANG II-induced VAFs and confirmed that HSYA inhibited NLRP3 inflammasome assembly and activation (Figure 2). A chronic inflammatory response is considered the primary feature of vascular remodeling, and the NLRP3 inflammasome plays a vital role in vascular disease [32]. NLRP3, the upstream molecule of the NF-κB signaling pathway, could be activated with vascular injury. Activated NLRP3 links to the PYD domains of ASCs through their N-terminal hemoprotein domains and then recruit pro-Caspase1 to form an NLRP3 inflammasome. Pro-Caspase1 becomes active Caspase1 via cutting off the p20/10 subunit after assembly. Activated Caspase1 promotes conversion of pro-IL18 and pro-IL1β to IL-18 and IL-1β, respectively; these are released to extracellular space and cause inflammatory responses [33]. When VAFs were irritated using ANG II, the expressions of NLRP3-inflammasome-related proteins such as NLRP3, ASC, and Caspase1 were significantly upregulated; this was reversed with HSYA in a dose-dependent manner (Figure 2). Moreover, in the HSYA groups, the level of pNF-κB in the nucleus decreased significantly due to the inhibition of TLR4/NF-κB signaling pathway activation.

NF-κB mediates inflammatory responses and upregulates expressions of a variety of pro-inflammatory molecules. The NLRP3 inflammasome, stimulated by NF-κB, promotes IL-1β release, prolongs inflammatory responses, and facilitates vascular remodeling through induction of VAF migration and differentiation [18]. In the present study, HSYA inhibited NF-κB phosphorylation and downregulated the expressions of cytokines such as TNFα, IL-1β, IL-6, and IL18 in the VAFs induced by ANG II. Elevated TNFα levels further elevated the expression of IL-6 and increased the level of vascular inflammation [34]. Thus, anti-inflammatory treatment that targets NLRP3 could reduce VAF migration and vascular remodeling. Furthermore, HSYA significantly inhibited VAF migration promoted by LPS and ANG II via NLRP3 inflammasome assembly limitation and activation (Figure 3). Our data suggested that ANG II and LPS could promote VAF migration by activating the NLRP3 inflammasome via the TLR4/NF-κB signaling pathway, which could be reversed with HSYA in a dose-dependent manner. However, in the current study, whether HSYA directly binds to TLR4 or indirectly affects the TLR4/NF-κB signaling pathway was not determined.

Autophagy is an autonomous protective mechanism in cells; it can degrade organelles or cytoplasmic proteins of diseases or senescence in cytoplasm to maintain the stability of the intracellular environment. Previous studies have indicated that autophagy is extremely important to cardiovascular-homeostasis maintenance [14]. However, excessive autophagy may disturb the internal environmental stability of vascular smooth-muscle cells and aggravate vascular remodeling and dysfunction of mesenteric arteries in hypertension [35]. Autophagy is negatively correlated with NLRP3 inflammasome activation. Autophagy activation remarkably downregulated expressions of NLRP3, IL-6, and IL-1β, whereas autophagy inhibition could increase the inflammatory responses of VAFs [36]. Ali et al. [37] showed that IIIM-941 could inhibit NLRP3 inflammasome activation by promoting autophagy in J774A.1 cells. In this study, we discovered that HSYA could also inhibit NLRP3 inflammasome activation. Our previous study suggested that blocking the autophagy process could promote VAF migration [20]. Thus, it is reasonable to hypothesize that HSYA inhibits NLRP3 inflammasome activation through autophagy regulation. To verify our hypothesis, we stimulated VAFs with rapamycin, a well-known autophagy activator. The results thereof showed that HSYA and rapamycin enhanced the mild fluorescence of autophagy spots in ANG II-induced VAFs, further confirming that autophagy activation could inhibit ANG II-induced VAF migration (Figure 4). Moreover, autophagy activation could inhibit NLRP3 inflammasome assembly and activation; this is consistent with previous results. However, the way that HSYA activates autophagy was not determined in this study; this requires further investigation. In addition, in vivo experiments should be carried out in the future to determine whether HSYA could exert the role of inhibiting NLRP3 inflammasome activation via autophagy promotion to improve vascular remodeling.

**Figure 5:**
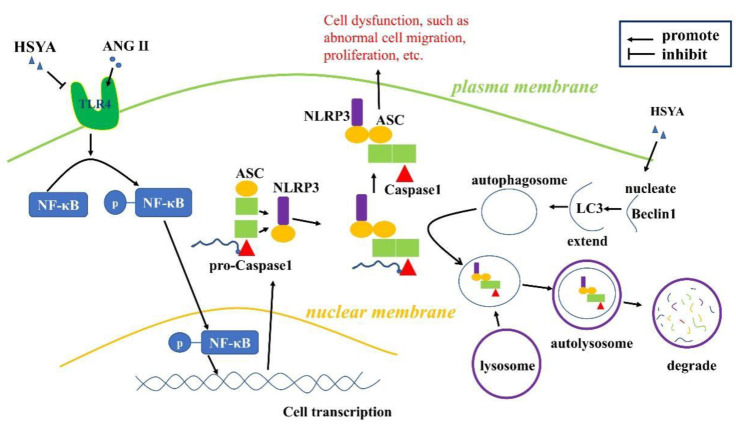
A proposed model that illustrates how HSYA induces autophagy and inhibits NLRP3 inflammasomes through the TLR4/NF-κB signaling pathway.

In conclusion, our results suggest that HSYA inhibits ANG II-induced VAF migration through autophagy activation and inhibition of NLRP3 inflammasome activation through the TLR4/NF-κB signaling pathway (Figure 5). Our study might provide an experimental basis for preclinical or clinical research regarding HSYA.

## 4. Materials and Methods

### 4.1. Materials

Hydroxysafflower yellow A (98%, HPLC; S26799) was purchased from Yuanye Biotechnology Co., Ltd. (Shanghai, China). Angiotensin II (98%, HPLC; A9290-10) was purchased from Solarbio Biotechnology Co., Ltd. (Beijing, China). Fetal bovine serum (FBS) was provided by Evergreen Biotechnology Co., Ltd. (Beijing, China). DMEM/F12 medium was provided by Gibco Life Technologies (Invitrogen, Carlsbad, CA, USA).

### 4.2. Protocol for Primary Cell Culture

Twenty 8-week-old Sprague–Dawley rats were purchased from Hebei Medical University (Shijiazhuang, Hebei province, China). After being fasted for 12 h, the animals were anesthetized with 3% pentobarbital sodium, and thoracic incision was made. Then, each thoracic aorta was separated under aseptic conditions. After the perivascular adipose tissue, the vascular intima, and the media of each animal were removed, each adventitia was cut into 1 mm^2^ pieces and put in a sterile Petri dish. Then, the adventitia tissue pieces were immersed in 10 mL of a growth medium (15% FBS + 85 % DMEM/F12 medium) and maintained at the temperature of 37 °C in a 5% CO_2_ incubator. When the degree of cell fusion around the tissue reached more than 90%, cells were transferred from the P0 generation to P1 generation. P3-generation cells were used in the experiment.

### 4.3. Dose–Effect Relationship and Time–Effect Determination of ANG II and HSYA

The VAFs were seeded into a 96-well plate and treated with different concentration gradients of ANG II (0, 10^−5^, 10^−6^, 10^−7^, 10^−8^, or 10^−9^ mol·L^−1^) or HSYA (0, 5, 10, 20, 40, 80, or 160 μmol·L^−1^), and the blank control group (pure medium without cells) was treated with neither ANG II nor HSYA. Before stimulation with ANG II or HSYA, the cells were cultured serum-free for 12 h. Then, 100 μL of medium containing the corresponding concentration of ANG II or HSYA was added to each group, and the cells were cultured for another 24 h. Regarding the determination of the treatment times with ANG II and HSYA, cells were treated for different time gradients of ANG II or HSYA (0 h, 12 h, 24 h, and 48 h). ANG II or HSYA was added in a reverse time order. After the last administration of ANG II or HSYA, which was considered to be at 0 h, each absorbance value was measured. CCK8 (Shanghaishare-bioBiotechnology Co., Ltd., Shanghai, China) was used to measure absorbance and calculate cell viability. This experiment was repeated three times in parallel.

### 4.4. Cell Treatment

The cells were divided into a control group (the NC group), an ANG II group, a HSYA20 group, an HSYA40 group, and an HSYA60 group. Cell treatment was as follows: The cells in the NC group were cultured normally, and the cells in the other four groups were first stimulated with 10^−7^ mol·L^−1^ of ANG II for 30 min to construct the ANG II-induced VAF migration model. After successful modeling, a medium that contained 20 μmol·L^−1^ of HSYA, 40 μmol·L^−1^ of HSYA, or 60 μmol·L^−1^ of HSYA was added to the cells, and the cells were cultured for 24 h. Cells stimulated with LPS were divided into a control group (the NC group), an ANG II group, an HSYA group, an LPS group, and an HSYA + LPS group. After 30 min of ANG II treatment, the cells were cultured with a medium that contained 40 μmol·L^−1^ of HSYA, 100 nmol·L^−1^ of LPS, or 40 μmol·L^−1^ of HSYA + 100 nmol·L^−1^ of LPS for 24 h.

### 4.5. Wound-Healing Assay

To carry out the wound-healing assay, cells were seeded in 6-well plates at a density of 2 × 10^5^ cells per milliliter and serum-starvation cultured for 24 h. Wounds were made across the cell plates using a sterile plastic tip and washed with PBS. Then, VAFs were cultured with a fresh medium alone or with a medium that contained ANG II (10^−7^ mol·L^−1^) or HSYA at various concentrations (20 μmol·L^−1^, 40 μmol·L^−1^, or 60 μmol·L^−1^) for 24 h. Cell migration was monitored under a phase-contrast microscope (Olympus Optical Co., Tokyo, Japan) at 0 and 24 h. The VAFs were treated with ANG II, HSYA, or LPS as above to confirm the inhibitory effect of HSYA on the NLRP3 inflammasome in VAF migration. To further confirm the role of HSYA in autophagy, the VAFs were pretreated with Rapamycin (100 nM) for 30 min, then treated with ANG II (10^−7^ mol·L^−1^) and HSYA (40 μmol·L^−1^) for 24 h. Cell migration was calculated using the following formula:

Migration rate = (0 h wound width − 24 h wound width)/0 h wound width

All experiments were performed in triplicate and repeated at least three times.

### 4.6. Double Immunofluorescence Staining

An analysis of the coexpression of Caspase1, ASC, and NLRP3 in the VAFs was performed using double immunofluorescence staining. Briefly, VAFs in different groups were each fixed with 4% paraformaldehyde and washed 3 times with PBS. After being treated with 0.25% Triton × 100 and goat serum, the VAFs were incubated with the required primary antibodies at 4 °C for 12 h. Then, the protein–primary antibody complex was recognized with secondary antibodies that were conjugated to fluorescein. The primary antibodies used in this study include Caspase1, NLRP3, and ASC (Abway Technology, Shanghai, China). The fluorescent secondary antibodies used in this study include Alexa Fluor 549 for the primary antibody of Caspase1 and Alexa Fluor-488 for the primary antibodies of NLRP3 and ASC (Abway Technology, Shanghai, China). The cells were incubated with an anti-fluorescence quenching agent that contained DAPI and were observed under a microscope.

### 4.7. MDC Assay

Autophagic vacuoles were labeled with monodansylcadaverin (MDC, KeiGEN Biotechnology, Nanjing, China). The cells were seeded in a 24-well plate and pretreated with rapamycin for two hours, followed by treatment with ANG II and HSYA for 24 h. After that, MDC dye was applied for visualization of autophagic vacuoles.

### 4.8. Western Blot Assay

Cells were collected and homogenized in a radioimmunoprecipitation buffer (RIPA). Then, a BCA protein assay kit (Pierce, Rockford, IL, USA) was used for protein-content determination. Identical amounts of proteins were loaded and separated via 8–12.5% SDS-PAGE gel electrophoresis and then transferred to polyvinylidene fluoride membranes (PVDF) (Applygen Technologies, Beijing, China). After being blocked with 20% skimmed milk, the proteins were identified via the primary antibodies. Then, the protein–antibody complex was incubated with secondary-antibodies that were conjugated to horseradish peroxidase. ECL luminescent solution (ThermoFisher Scientific, Shanghai, China) was used for development of blots composed of the protein–primary antibody–secondary antibody complex. Primary antibodies used in this study included NLRP3, ASC, Caspase 1, TLR4, NF-κB, p-NF-κB, LC3, Beclin1 (Abway Technology, Shanghai, China), and GAPDH (Cell Signaling Technology, Danvers, MA, USA). The quantification of the gray values of the blots was performed using Image J software (National Institutes of Health, Bethesda, MD, USA).

### 4.9. RNA Isolation and Quantitative Real-Time PCR 

The total RNA was extracted from the VAFs using a Trizol reagent (Invitrogen, Carlsbad, CA, USA). Then, an RT kit (Invitrogen, Carlsbad, CA, USA) was used for first-strand cDNA generation. Quantitative real-time PCR was performed using the primers listed in Supplementary Table S1. Applied Biosystems and Quant Studio™ Design & Analysis Software (Life Technologies, San Francisco, CA, USA) were applied for amplifications in 35 cycles, with SYBR green fluorescence (TransGene Biotech, Beijing, China). The samples were quantitated using the comparative CT method normalized to GAPDH.

### 4.10. Statistics Analysis

Statistical analysis was performed using a one-way ANOVA (Dunnett’s *t*-test) and a two-tailed Student’s *t*-test with GraphPad Prism 5.0 (GraphPad Software, San Diego, CA, USA). The data in this study are shown as means ± SEM. Results were considered statistically significant at *p* < 0.05.

## Data Availability

The data presented in this study are available in the article.

## Supplementary Materials

The supporting information can be downloaded at: https://www.mdpi.com/article/10.3390/ijms24010172/s1.
