# Supplementary material for: Hydroxysafflower Yellow A Inhibits Vascular Adventitial Fibroblast Migration via NLRP3 Inflammasome Inhibition through Autophagy Activation
Source: Int J Mol Sci. 2022 Dec 22;24(1):172. doi: 10.3390/ijms24010172 (PMC9820330; doi:10.3390/ijms24010172)
Supplement: Supplementary file 1 [file ijms-24-00172-s001.zip › ijms-2071520-supplementary.pdf]

Supplementary Table S1: A list of primers for qPCR

Gene	Forward	Reverse
Gapdh	AGTGCCAGCCTCGTCTCATA	TGAACTTGCC-GTGGGTAGAG
IL-1β	GACTTCACCATGGAACCCGT	GGAGACTGCC-CATTCTCGAC
IL-6	GAGACTTCCAGCCAGTTGCC	TGAAGTCTCCTCTCCGGACTT
Tnfa	ATTGTGGCTCTGGGT-CCAAC	AGCGTCTCGTGTGTTTCTGA
IL-18	CAGCCAACGAATCCCAGACC	ACAGATAGGGTCACAGCCAG

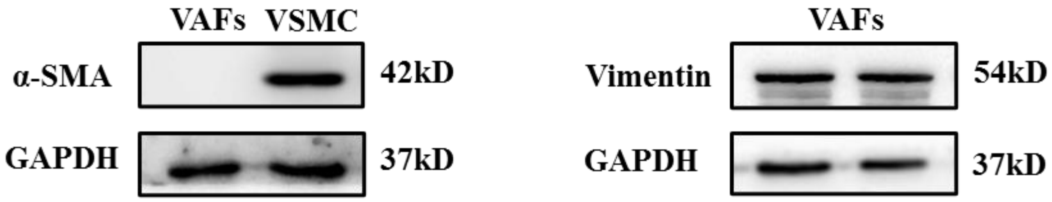

Supplementary Figure S1. Western-blot analysis of α-SMA or vimentin.

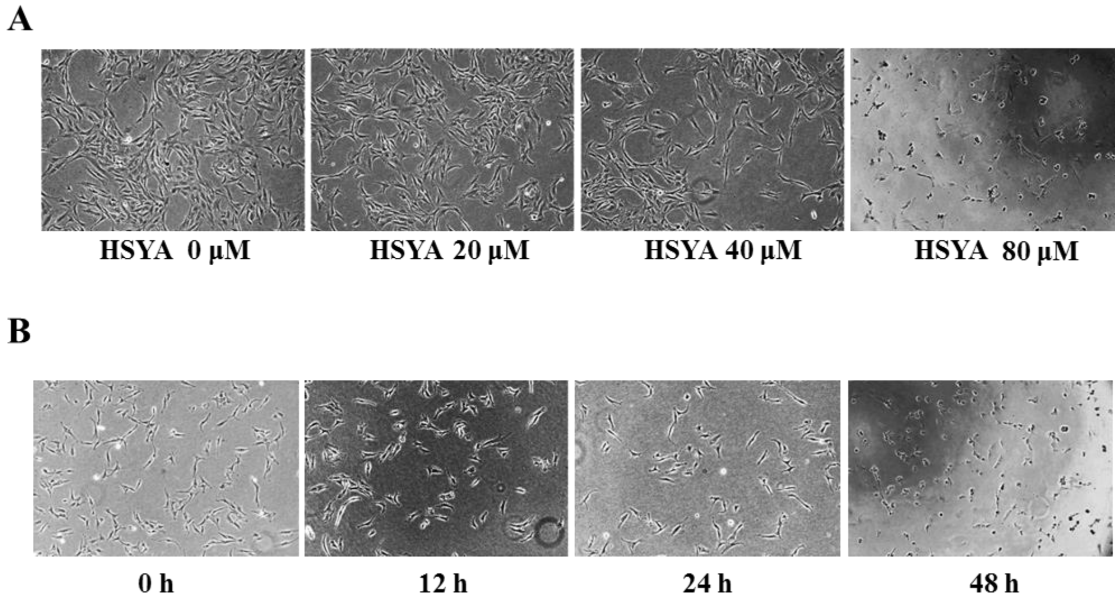

Supplementary Figure S2. Morphology of VAFs treated with HSYA. (**A**) Morphology of VAFs treated with different concentration of HSYA. (**B**) Morphology of VAFs treated with different time of HSYA at the concentration of 40 μM.

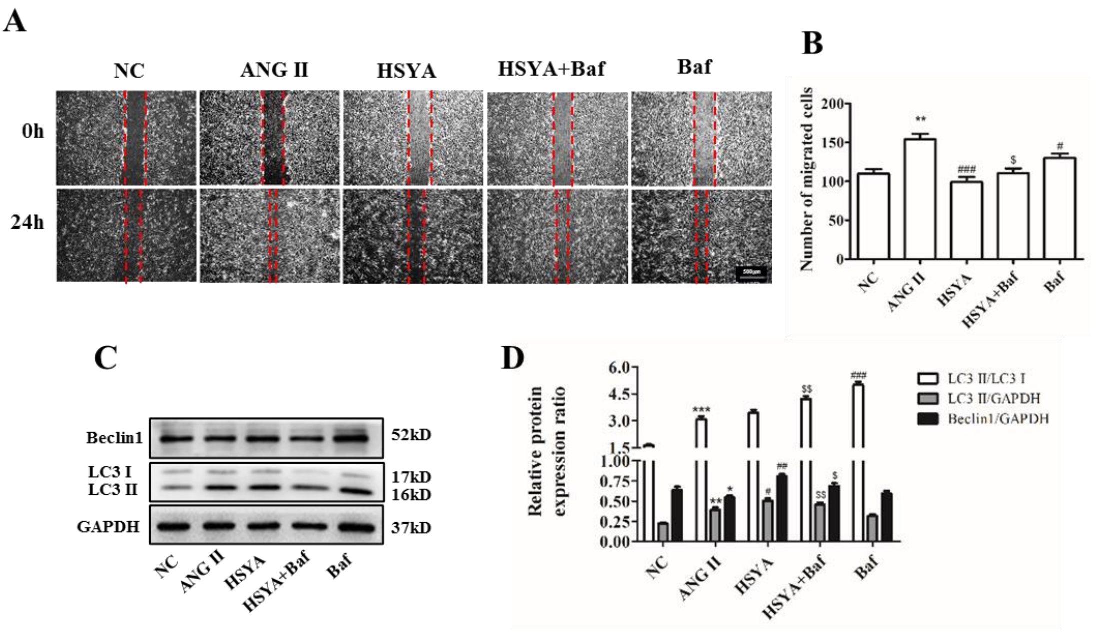

Supplementary Figure S3. HSYA inhibits VAFs migration by promoting autophagy. **(A**) Wound healing assay of VAFs treated with or without ANG II, HSYA and Baf (n=3). Scale bar, 500 μm. (**B**) The quantification of mobility of VAFs in the five groups of Figure. 3A. (**C**) Western-blot analysis of P62, Beclin1 and LC3 (n=4). (**D**) The quantification of protein blots of Figure. 3C. Compared with NC group, # compared with ANG II group, $ compared with LPS group, $^{*/\#/\$}P < 0.05$, $^{**/\#\#/\$\$}P < 0.01$, $^{***/\#\#\#/\$\$\$}P<0.001$.
